# Supplementary material for: Reproducible, data-driven characterization of sleep based on brain dynamics and transitions from whole-night fMRI
Source: medRxiv. 2024 Apr 24:2024.04.24.24306208. Preprint. [Version 1] doi: 10.1101/2024.04.24.24306208 (PMC11188122; doi:10.1101/2024.04.24.24306208)
Supplement: Supplementary file 1 [file media-1.docx]

Reproducible data-driven characterization of sleep from whole-night fMRI

Fan Nils Yang, Dante Picchioni, Jacco A. de Zwart, Yicun Wang, Peter van Gelderen, Jeff H. Duyn

Advanced MRI Section, Laboratory of Functional and Molecular Imaging, National Institute of Neurological Disorders and Stroke, National Institutes of Health, Bethesda, MD, USA

# Supplement

**Supplementary Figure 1.** The mean Lifetime of 21 HMM states. The HMM states are organized based on the results of modular analysis. The error bars represent the standard error of the mean. 

**Supplementary Figure 2.** Physiological variables associated with each HMM state during Night 2. The error bars represent the standard error of the mean. Panel **A**: slow wave density (the percentage of TR that had slow waves); Panel **B**: variation in PPG amplitude (z-score); Panel **C**: variation in Respiratory Volume per Time (z-score); Panel **D**: variation in Heart Rates; Panel **E**: time within a fMRI Run (TR, 3-second); Panel **F**: time since experiment start (TR, 3-second).

**Supplementary Figure 3.** Physiological variables associated with each HMM state during Night 1. The error bars represent the standard error of the mean. Panel **A**: slow wave density (the percentage of TR that had slow waves); Panel **B**: variation in PPG amplitude (z-score); Panel **C**: variation in Respiratory Volume per Time (z-score); Panel **D**: variation in Heart Rates; Panel **E**: time within a fMRI Run (TR, 3-second); Panel **F**: time since experiment start (TR, 3-second).

**Supplementary Figure 4.** Mean fMRI activation (percent signal change) for each state relative to baseline averaged over all HMM states. Notes: Un: Undefined Network; DMN: Default Mode Network; VIS: Visual Network; FPN: Frontoparietal Network; REW: Reward Network; DAN: Dorsal Attention Network; VAN: Ventral Attention Network; SAL: Salience Network; CON: Cingulo-Opercular Network; dSMN: Somatomotor Dorsal Network; lSMN: Somatomotor Lateral Network; AUD: Auditory Network; PMN: ParietoMedial Network; MTL: Medial Temporal Network; pHIP: Posterior Hippocampus; BG: Basal Ganglia; THAL: Thalamus; CB: Cerebellar Cortex.

**Supplementary Figure 5.** FC Patterns for each state relative to baseline averaged over all HMM states. HMM states were color-coded based on modules (Green: light N2 module; Dark Blue: N3 module; Light Blue: deep N2 module; Orange: REM module; Yellow: Wake Module). Notes: Y-axis from top to bottom or X-axis from left to right: Un: Undefined Network; DMN: Default Mode Network; VIS: Visual Network; FPN: Frontoparietal Network; REW: Reward Network; DAN: Dorsal Attention Network; VAN: Ventral Attention Network; SAL: Salience Network; CON: Cingulo-Opercular Network; dSMN: Somatomotor Dorsal Network; lSMN: Somatomotor Lateral Network; AUD: Auditory Network; PMN: ParietoMedial Network; MTL: Medial Temporal Network; pHIP: Posterior Hippocampus; BG: Basal Ganglia; THAL: Thalamus; CB: Cerebellar Cortex.

**Supplementary Figure 6.** Correlation matrix between FC patterns of each pair of HMM states. The color bar represents Pearson correlation coefficients. The figure depicts similar modules as the results of the modular analysis in **Figure 3**. For example, states within the Deep-N2 module (states 1, 2, 3, 12, and 17) are highly correlated to each other. These states also show a higher similarity with the N3 module compared to the states within the Light-N2 module (states 5, 7, 9, 18, 21, and 15, except for state 14, which might be due to high physiological variation associated with state 14, see **Supplementary Figure 2 B&D**). REM states 6 and 19 correlated with each other. States within the Wake module (11, 13, 16, and 20) highly correlated with each other. 

**Supplementary Figure 7.** Model evaluation parameters. The error bars represent the standard error of the mean. Panel **A**: free energy; Panel **B**: maximum Occupancy (percentage); Panel **C**: median Occupancy (percentage); Panel **D**: Wilk's Λ; Panel **E**: mean HMM state Lifetime (TR, 3-second).

**Supplementary Figure 8.** State timecourse of HMM states and its associations with PSG stages, variation in PPG amplitude, and variations in RespRVT signals of an example run.

**Supplementary Figure 9.** State timecourse of HMM states and its associations with PSG stages, variation in PPG amplitude, and variations in RespRVT signals of a second example run.
